# Serum uric acid to creatinine ratio and long-term target vessel events in diabetes patients undergoing PCI with drug-eluting stents implantation: a retrospective study

**DOI:** 10.3389/fendo.2025.1599158

**Published:** 2025-06-27

**Authors:** Penghui Cui, Shuting Wang, Zherui Zhang, Ruihua Wang

**Affiliations:** ^1^ Department of Cardiology, Changzhi People’s Hospital, Changzhi, China; ^2^ Department of Endocrinology, Changzhi Medical College, Changzhi, China; ^3^ School of Public Health, Jilin University, Changchun, China

**Keywords:** diabetes mellitus, coronary heart disease, target vessel events, serum uric acid to creatinine ratio, percutaneous coronary intervention

## Abstract

**Aim:**

This study aimed to investigate the association between the serum uric acid to creatinine ratio (SUA/Cr) and long-term target vessel events (TVEs) in diabetes mellitus (DM) patients undergoing percutaneous coronary intervention (PCI) with drug-eluting stents (DES).

**Methods:**

From July 2009 to August 2011, a total of 2533 patients with coronary heart disease (CHD) who underwent PCI with DES implantation were enrolled to evaluate the relationship between the SUA/Cr and TVEs during a median follow-up of 29.8 months. Multivariable logistic regression and restricted cubic spline analyses were performed, and subgroup analyses were conducted to explore potential effect modifiers.

**Results:**

The TVEs were significantly associated with previous male gender (OR=1.58, 95%CI: 1.07~2.32, p=0.021), PCI (OR=3.58, 95% CI: 2.27~5.67, p<0.001),previous stroke(OR=2.19,95%CI:1.24~3.89,p=0.007),triglyceride(OR=1.15, 95%CI:1.06~1.26, p=0.002), length of stent (OR=1.01, 95%CI:1~1.01, p<0.001), and diameter of stent (OR=0.62,95%CI:0.41~0.92,p=0.019). In DM patients, Multivariable logistic regression analyses revealed that higher SUA/Cr was independently associated with a reduced risk of TVEs (adjusted OR=0.72, 95% CI: 0.53–0.97, p=0.031). Restricted cubic spline analysis confirmed a linear inverse relationship between SUA/Cr and TVEs (p for non-linearity=0.782). Subgroup analyses revealed stronger protective effects in non-smokers and non-ST elevation acute coronary syndrome (NSTE-ACS) patients within the DM cohort.

**Conclusion:**

A higher SUA/Cr is independently associated with a reduced risk of TVEs in DM patients undergoing PCI with DES. SUA/Cr holds promise as a potential prognostic biomarker for risk stratification in DM patients undergoing PCI.

## 1 Introduction

Diabetes mellitus (DM) is an escalating global health challenge and a well-established risk factor for coronary heart disease (1). Its rising prevalence has led to a growing burden of cardiovascular complications. Compared to non-DM individuals, DM patients undergoing percutaneous coronary intervention (PCI) face a significantly higher incidence of postoperative target vessel events (TVEs), including target vessel revascularization (TVR) and in-stent restenosis (ISR), resulting in poorer long-term outcomes (2). Given these risks, the identification of reliable biomarkers for more precise risk stratification and improved post-PCI management in DM patients is of critical clinical importance.

Serum uric acid (SUA), the end product of purine metabolism, has long been implicated in the pathogenesis of cardiovascular events (3, 4) However, its role as an independent predictor of cardiovascular risk remains controversial (5–7). Some studies suggest that elevated SUA levels contribute to an increased risk of atherosclerosis and cardiovascular events by inducing oxidative stress, inflammation, and endothelial dysfunction (8). Conversely, other research indicates that uric acid may exert antioxidant properties under certain conditions, offering potential protective effects on the cardiovascular system (9–11). Given these conflicting findings, relying solely on SUA as a cardiovascular risk marker may be inherently limited. Since SUA levels are largely influenced by renal excretion, recent studies have increasingly focused on renal function-normalized SUA, expressed as the serum uric acid to creatinine ratio (SUA/Cr), as a more robust biomarker. Emerging evidence suggests that SUA/Cr is closely associated with metabolic disorders and plays a crucial role in predicting the progression and prognosis of acute myocardial infarction (AMI), heart failure, hypertension, diabetes, hypertensive disorders of pregnancy, ischemic stroke, and fatty liver disease (5, 12–20). On the other hand, there is a lack of data on the clinical value of SUA/Cr in patients with diabetes undergoing PCI, and few published large-scale validation studies have included this population. In addition, the vast majority of available research have concentrated either upon the independent effects of SUA or Cr on long-term outcomes in DM patients with coronary artery disease (CAD) while the possible prognostic value of SUA/Cr as an independent risk predictor was less extensively explored.

Although recent studies, such as the URRAH project by D’Elia et al. (12), have suggested a potential association between SUA/Cr and cardiovascular mortality in diabetic individuals, these studies primarily focused on all-cause or cardiovascular mortality without evaluating PCI-specific outcomes. Similarly, Casiglia et al. (21) reported prognostic cut-off values for SUA/Cr in general cardiovascular events but did not explore post-PCI restenosis or revascularization events. Importantly, to our knowledge, no prior large-scale study has comprehensively evaluated the prognostic significance of SUA/Cr on long-term TVEs, specifically in the high-risk diabetic population undergoing PCI with drug-eluting stents (DES). Thus, our study fills this critical gap by providing focused insights into the association between SUA/Cr and post-PCI vascular outcomes.

Therefore, the aim of this study was to examine the potential association between SUA/Cr and the risk of long-term TVEs in patients with DM undergoing PCI with DES implantation. Our prespecified hypothesis was that a higher SUA/Cr ratio would be independently associated with an increased risk of long-term TVEs, offering novel insights into cardiovascular risk stratification for DM patients.

## 2 Materials and methods

### 2.1 Study design and population

This retrospective cohort study utilized a publicly available dataset from patients who underwent PCI with drug-eluting stents (DES) at the First Affiliated Hospital of Zhengzhou University, Zhengzhou, China, between July 2009 and August 2011. The original cohort comprised 2,533 patients diagnosed with coronary artery disease (CAD).

After applying exclusion criteria—patients with implausible values (n = 2), incomplete data (n = 422), and outliers in the serum uric acid to creatinine ratio (SUA/Cr, below the 0.5th or above the 99.5th percentile, n = 22)—a final cohort of 2,087 patients was included in the analysis.

All patients underwent PCI following standard protocols, with a median follow-up duration of 29.8 months (interquartile range: 25.6–34.0 months). The dataset was accessed from the Dryad repository (https://doi.org/10.5061/dryad.13d31) and was originally published by Yao et al. (22).

**Figure 1:**
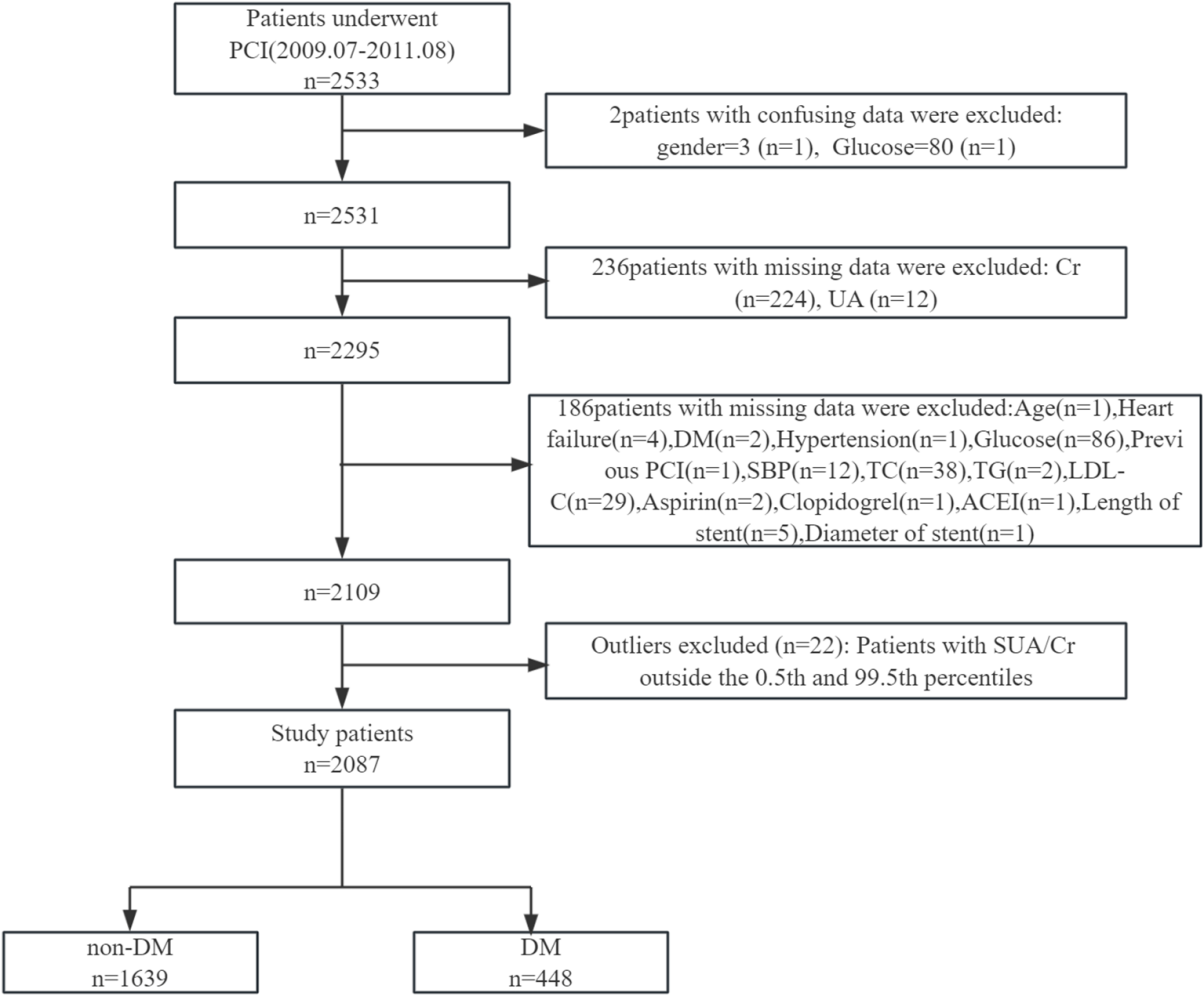
Flowchart of participant selection. PCI, percutaneous coronary intervention; Cr, creatinine; UA, uric acid; DM, diabetes mellitus; SBP, systolic blood pressure; TC, total cholesterol; TG, triglyceride; LDL-C, low-density lipoprotein cholesterol; ACEI, angiotensin converting enzyme inhibitor.

This study adheres to the Declaration of Helsinki and received a waiver for informed consent due to the secondary use of publicly available data. A detailed flowchart of participant selection is provided in **Figure 1**.

### 2.2 Data collection

Data collection encompassed baseline demographics, clinical characteristics, comorbidities, laboratory test results, procedural details, and follow-up information. These data were recorded electronically and obtained from various sources, including hospital electronic medical records, medical insurance data, telephone interviews, and in-person follow-ups at the research site.

### 2.3 Definitions

The SUA/Cr was calculated as the ratio of SUA (µmol/L) to serum creatinine (µmol/L). Smoking history was defined as any smoking within the past 10 years. DM was defined as a fasting plasma glucose level >6.1 mmol/L, hemoglobin A1c >6.5%, or current treatment with insulin or oral hypoglycemic agents. Hypertension was defined as systolic blood pressure (SBP) ≥140 mmHg, diastolic blood pressure (DBP) ≥90 mmHg, or the use of antihypertensive medications. The estimated glomerular filtration rate (eGFR) was calculated using the modified MDRD equation: eGFR=175×SCr^−1.234^×Age^−0.179^×0.79 (if female), where SCr is serum creatinine in mg/dL, Age is in years (23). TVR was defined as any repeat revascularization, either PCI or coronary artery bypass graft (CABG), within the target vessel. ISR was defined as ≥50% diameter stenosis within the stented segment, as identified by follow-up coronary angiography.

### 2.4 Study outcomes

For cardiovascular disease events, an independent clinical endpoint committee adjudicated all outcomes strictly according to established guidelines and contemporary diagnostic criteria. The primary endpoint of TVE was defined as a composite of TVR and ISR, ensuring precise and standardized outcome assessment.

### 2.5 Statistical analysis

Continuous variables were expressed as mean ± standard deviation (SD) for normally distributed data or median with interquartile range (IQR) for skewed data. Categorical variables were presented as frequencies and percentages. Differences between groups were assessed using the chi-square test for categorical variables, one-way ANOVA for normally distributed continuous variables, and the Kruskal-Wallis test for skewed continuous variables.

Univariate and multivariate logistic regression analyses were conducted to evaluate the association between SUA/Cr and TVEs. Covariates for the multivariate models were selected based on a univariate p-value <0.1 or clinical relevance. Clinically important variables, even if not statistically significant, were included based on prior research and clinical judgment. The restricted cubic splines were used to assess whether there was a linear or nonlinear correlation between SUA/Cr and TVEs in patients with DM (with a threshold of P < 0.10).

To minimize the influence of extreme outliers, we focused on the central 99% of the data by excluding values of the SUA/Cr below the 0.5th percentile and above the 99.5th percentile. This method, implemented using the quantile function in R, is a common practice in clinical research to reduce the impact of extreme values and enhance the reliability of findings. This approach is supported by previous studies that emphasize the importance of outlier removal to improve statistical robustness in clinical data analysis (24, 25).

All the analyses were performed with the statistical software packages R 3.3.2 (http://www.R-project.org, The R Foundation) and Free Statistics software versions 1.9.2.A two-tailed test was performed, and p < 0.05 was considered statistically significant.

## 3 Results

### 3.1 Characteristics of the study population by diabetes mellitus

Among the 2,087 participants, 448 (21.5%) had DM). Compared to non-DM patients, DM patients were older (61.79 ± 10.27 vs. 59.39 ± 11.25 years, p < 0.001) and had a higher prevalence of hypertension (63.4% vs. 46.4%, p < 0.001). DM patients were also less likely to smoke (23.2% vs. 35.6%, p < 0.001). The general baseline data of the study subjects are shown in **Table 1**.

**Table 1:** Baseline characteristics of the study population by DM.

Characteristics	Overall	non-DM	DM	*P-*value
N	2087	1639	448	
Demographics				
Male, n (%)	1415 (67.8)	1139 (69.5)	276 (61.6)	0.002
Age,years	59.90 ± 11.09	59.39 ± 11.25	61.79 ± 10.27	< 0.001
Clinical presentation, n (%)				0.981
STEMI	521 (25.0)	410 (25)	111 (24.8)	
NSTE-ACS	1273 (61.0)	998 (60.9)	275 (61.4)	
SA	293 (14.0)	231 (14.1)	62 (13.8)	
SBP, mmHg	102.69 ± 28.58	103.46 ± 28.59	99.91 ± 28.42	0.020
DBP, mmHg	77.09 ± 12.01	77.05 ± 12.15	77.23 ± 11.50	0.771
Medical history,n (%)				
Heart failure	243 (11.6)	181 (11)	62 (13.8)	0.102
Atrial fibrillation	41 (2.0)	28 (1.7)	13 (2.9)	0.107
Cardiac shock	2 (0.1)	2 (0.1)	0 (0)	1.000
Previous AMI	205 (9.8)	159 (9.7)	46 (10.3)	0.721
Previous stroke	107 (5.1)	76 (4.6)	31 (6.9)	0.052
Previous PCI	137 (6.6)	102 (6.2)	35 (7.8)	0.229
Hypertension	1045 (50.1)	761 (46.4)	284 (63.4)	< 0.001
Smoking	688 (33.0)	584 (35.6)	104 (23.2)	< 0.001
Laboratory data				
Cr, µmol/L	72.00 ± 26.07	72.12 ± 24.16	71.56 ± 32.11	0.686
UA, µmol/L	303.69 ± 88.40	306.71 ± 88.88	292.62 ± 85.81	0.003
Glucose, mmol/L	5.95 ± 2.58	5.42 ± 2.03	7.91 ± 3.35	< 0.001
TG, mmol/L	1.6 (1.1, 2.3)	1.5 (1.1, 2.2)	1.8 (1.3, 2.6)	< 0.001
TC, mmol/L	4.26 ± 1.06	4.26 ± 1.07	4.26 ± 1.04	0.952
HDL-C, mmol/L	1.06 ± 0.31	1.07 ± 0.32	1.02 ± 0.31	0.002
LDL-C, mmol/L	2.67 ± 0.93	2.69 ± 0.95	2.62 ± 0.89	0.171
Treatment, n (%)				
Aspirin	2063 (98.9)	1623 (99)	440 (98.2)	0.154
Clopidogrel	2000 (95.8)	1569 (95.7)	431 (96.2)	0.233
β-blocker	1468 (70.3)	1156 (70.5)	312 (69.6)	0.715
ACEI	1144 (54.8)	880 (53.7)	264 (58.9)	0.048
CCB	507 (24.3)	382 (23.3)	125 (27.9)	0.044
Statin	1958 (93.8)	1543 (94.1)	415 (92.6)	0.240
Radial artery acess, n (%)	2038 (97.7)	1600 (97.6)	438 (97.8)	0.855
Number of diseased vessels, n (%)				
1-diseased vessel	813 (39.0)	679 (41.4)	134 (29.9)	< 0.001
2-diseased vessels	777 (37.2)	607 (37)	170 (37.9)	0.724
≥3-diseased vessels	492 (23.6)	350 (21.4)	142 (31.7)	< 0.001
Location of target lesions, n (%)				
LM	65 (3.1)	49 (3)	16 (3.6)	0.530
LAD	1720 (82.4)	1335 (81.5)	385 (85.9)	0.027
LCX	1018 (48.8)	776 (47.3)	242 (54)	0.012
RCA	1030 (49.4)	772 (47.1)	258 (57.6)	< 0.001
Characteristics of lesions, n (%)				
Occulsion	273 (13.1)	208 (12.7)	65 (14.5)	0.312
CTO	183 (8.8)	138 (8.4)	45 (10)	0.281
Ostio lesion	229 (11.0)	188 (11.5)	41 (9.2)	0.164
Bifurcation lesion	363 (17.4)	294 (17.9)	69 (15.4)	0.210
Restenosis	26 (1.2)	21 (1.3)	5 (1.1)	0.780
Number of treated vessels, n (%)				0.006
1-treated vessel	1209 (57.9)	979 (59.7)	230 (51.3)	
2-treated vessels	696 (33.3)	524 (32)	172 (38.4)	
≥3-treated vessels	182 (8.7)	136 (8.3)	46 (10.3)	
Number of stents, n (%)				0.002
1	815 (39.1)	673 (41.1)	142 (31.7)	
2	614 (29.4)	475 (29)	139 (31)	
3	354 (17.0)	269 (16.4)	85 (19)	
≥4	304 (14.6)	222 (13.5)	82 (18.3)	
Length of stent, mm	50.07 ± 32.69	48.66 ± 32.37	55.23 ± 33.40	< 0.001
Diameter of stent, mm	3.10 ± 0.94	3.14 ± 1.04	2.97 ± 0.40	< 0.001

Data were mean ± SD or median (IQR) for skewed variables or numbers (proportions) for categorical variables.DM, diabetes mellitus; STEMI, ST-elevation myocardial infarction; NSTE-ACS, non-ST elevation acute coronary syndrome; SA, stable angina; SBP, systolic blood pressure; DBP, diastolic blood pressure; AMI, acute myocardial infarction; PCI, percutaneous coronary intervention; CABG, coronary artery bypass graft; Cr, creatinine; UA, uric acid; TG, triglyceride; TC, total cholesterol; HDL-C, high density lipoprotein cholesterol; LDL-C, low-density lipoprotein cholesterol; ACEI, angiotensin converting enzyme inhibitor; CCB, calcium channel blockers; LM, left main coronary artery; LAD, left anterior descending artery; LCX, left circumflex artery; RCA, right coronary artery; CTO, chronic total occlusions.

Laboratory results revealed that DM patients had higher fasting glucose (7.91 ± 3.35 vs. 5.42 ± 2.03 mmol/L, p < 0.001) and TG levels (1.8 [1.3–2.6] vs. 1.5 [1.1–2.2] mmol/L, p < 0.001). However, their SUA (292.62 ± 85.81 vs. 306.71 ± 88.88 μmol/L, p = 0.003) and HDL-C levels (1.02 ± 0.31 vs. 1.07 ± 0.32 mmol/L, p = 0.002) were lower.

Procedurally, DM patients had a higher proportion of multivessel disease (31.7% vs. 21.4%, p < 0.001) and received longer stents (55.23 ± 33.40 vs. 48.66 ± 32.37 mm, p < 0.001), but stent diameters were smaller (2.97 ± 0.40 vs. 3.14 ± 1.04 mm, p < 0.001).

### 3.2 Risk factors for TVEs in the overall population

As shown in **Table 2**, both univariate and multivariate analyses identified male gender, previous PCI, previous stroke, elevated triglyceride levels, longer stent length, and smaller stent diameter as significant risk factors for TVEs (all p < 0.05).In contrast, the SUA/Cr ratio was not significantly associated with TVEs in univariate analysis (OR = 0.95, 95% CI: 0.85–1.07, p = 0.404). Even after adjusting for potential confounders in multivariate analysis, SUA/Cr remained non-significant (OR = 0.95, 95% CI: 0.84–1.07, p = 0.385).

**Table 2:** Univariate and multivariate analysis for predictors of TVEs in overall population.

Variable	Univariate analysis		Multivariate analysis	
	OR (95% CI)	*P-*value	OR (95% CI)	*P-*value
SUA/Cr	0.95 (0.85~1.07)	0.404	0.95 (0.84~1.07)	0.385
Demographics				
Gender	1.58 (1.07~2.32)	0.021	1.54 (1.03~2.3)	0.034
Age	0.99 (0.98~1.01)	0.451		
Clinical presentation				
STEMI	1 (ref)			
NSTE-ACS	1.93 (1.21~3.06)	0.006		
SA	2.02 (1.12~3.63)	0.019		
SBP	1.01 (1~1.01)	0.020	1.01 (1~1.01)	0.016
DBP	1 (0.99~1.02)	0.770		
Medical history				
Heart failure	0.95 (0.56~1.61)	0.855		
Atrial fibrillation	1.39 (0.49~3.94)	0.540		
Previous AMI	1.09 (0.63~1.86)	0.762		
Previous stroke	2.19 (1.24~3.89)	0.007	1.93 (1.06~3.5)	0.030
Previous PCI	3.58 (2.27~5.67)	<0.001	3.57 (2.1~6.09)	<0.001
Hypertension	1 (0.72~1.39)	0.985		
DM	1.1 (0.74~1.63)	0.627	0.97 (0.64~1.46)	0.881
Smoking	1.28 (0.91~1.8)	0.158		
Laboratory data				
Glucose	1.03 (0.97~1.08)	0.310		
TC	1.13 (0.97~1.31)	0.106		
TG	1.15 (1.06~1.26)	0.002	1.18 (1.08~1.3)	<0.001
HDL-C	0.67 (0.38~1.17)	0.162		
LDL-C	1.05 (0.88~1.25)	0.588		
Treatment				
Aspirin	1.82 (0.24~13.54)	0.560		
Clopidogrel	0.72 (0.34~1.53)	0.394		
β-blocker	1.07 (0.74~1.55)	0.701		
ACEI	0.84 (0.6~1.16)	0.285		
CCB	0.96 (0.65~1.42)	0.856		
Statin	0.75 (0.4~1.39)	0.364		
Number of diseased vessels				
1-diseased vessel	0.78 (0.55~1.1)	0.157		
2-diseased vessels	0.98 (0.7~1.38)	0.918	0.96 (0.6~1.55)	0.873
≥3-diseased vessels	1.4 (0.97~2.01)	0.070	1.04 (0.54~2)	0.908
Location of target lesions				
LM	2.41 (1.2~4.82)	0.013	3.11 (1.49~6.49)	0.003
LAD	1.15 (0.73~1.8)	0.546		
LCX	1.22 (0.87~1.69)	0.248	0.86 (0.54~1.36)	0.512
RCA	1.29 (0.93~1.8)	0.131		
Characteristics of lesions				
Occulsion	0.83 (0.49~1.39)	0.472		
CTO	1.15 (0.66~2.01)	0.619		
Ostio lesion	0.88 (0.51~1.53)	0.651		
Bifurcation lesion	1.29 (0.86~1.94)	0.218	1.25 (0.82~1.91)	0.301
Restenosis	4.87 (2.01~11.77)	<0.001	1.27 (0.45~3.59)	0.655
Number of treated vessels				
1-treated vessel	1 (ref)			
2-treated vessels	1.36 (0.94~1.97)	0.103		
≥3-treated vessels	3.26 (2.06~5.17)	<0.001		
Number of stents				
1	1 (ref)			
2	1.25 (0.81~1.94)	0.313		
3	1.62 (1~2.63)	0.049		
≥4	2.43 (1.53~3.84)	<0.001		
Length of stent	1.01 (1~1.01)	<0.001	1.01 (1~1.01)	0.008
Diameter of stent	0.62 (0.41~0.92)	0.019	0.58 (0.37~0.92)	0.019

OR, odds ratio; CI, confidence interval; STEMI, ST-elevation myocardial infarction; NSTE-ACS, non-ST elevation acute coronary syndrome; SA, stable angina; SBP, systolic blood pressure; DBP, diastolic blood pressure; DM, diabetes mellitus; AMI, acute myocardial infarction; PCI, percutaneous coronary intervention; Cr, creatinine; UA, uric acid; TG, triglyceride; TC, total cholesterol; HDL-C, high density lipoprotein cholesterol; LDL-C, low-density lipoprotein cholesterol; ACEI, angiotensin converting enzyme inhibitor; CCB, calcium channel blockers; LM, left main coronary artery; LAD, left anterior descending artery; LCX, left circumflex artery; RCA, right coronary artery; CTO, chronic total occlusions.

### 3.3 Subgroup analyses

Subgroup analyses were performed to explore the association between SUA/Cr and TVEs across different strata, including DM), sex, age, and eGFR (**Table 3**).

**Table 3:** Subgroup analysis for association between SUA/Cr and TVEs.

Subgroups	n total	n event (%)	Unadjusted OR (95% CI)	Unadjusted *P*-value	Adjusted OR (95% CI)	Adjusted *P*-value	P for interaction
DM							0.043
No	1639	117 (7.1)	1.03 (0.91~1.16)	0.674	1 (0.87~1.13)	0.944	
Yes	448	35 (7.8)	0.7 (0.53~0.94)	0.016	0.74 (0.55~0.99)	0.046	
Gender							0.177
Female	672	36 (5.4)	0.85 (0.67~1.08)	0.178	0.81 (0.63~1.04)	0.097	
Male	1415	116 (8.2)	1.01 (0.89~1.15)	0.884	1 (0.88~1.15)	0.958	
Age, year							0.814
≥60	1124	77 (6.9)	0.92 (0.78~1.08)	0.326	0.93 (0.79~1.11)	0.438	
<60	963	75 (7.8)	0.98 (0.83~1.15)	0.784	0.94 (0.78~1.12)	0.463	
eGFR							0.856
≥60	1991	143 (7.2)	0.96 (0.85~1.08)	0.474	0.95 (0.84~1.08)	0.442	
<60	96	9 (9.4)	1.04 (0.52~2.11)	0.907	1.09 (0.31~3.8)	0.892	

OR, odds ratio; CI, confidence interval; DM, diabetes mellitus; eGFR, estimated glomerular filtration rate.

In the DM subgroup, SUA/Cr was significantly associated with a lower risk of TVEs (adjusted OR = 0.74, 95% CI: 0.55–0.99, p = 0.046). However, no significant association was observed in the non-DM subgroup (adjusted OR = 1.00, 95% CI: 0.87–1.13, p = 0.944), with a significant interaction effect (p for interaction = 0.043).

Stratified by sex, age, and eGFR categories, SUA/Cr showed no significant association with TVEs in females (adjusted OR = 0.81, 95% CI: 0.63–1.04, p = 0.097), males (adjusted OR = 1.00, 95% CI: 0.88–1.15, p = 0.958), or any subgroup (all p for interaction > 0.05).

### 3.4 Association of SUA/Cr with TVEs in DM patients

Multivariable logistic regression analyses demonstrated that higher SUA/Cr was significantly associated with a reduced risk of TVEs in DM patients (**Table 4**). The association remained consistent across all four models: Model 1 (OR = 0.72, 95% CI: 0.53–0.97, p = 0.031), Model 2 (OR = 0.72, 95% CI: 0.53–0.97, p = 0.030), Model 3 (OR = 0.73, 95% CI: 0.54–0.99, p = 0.040), and Model 4 (OR = 0.72, 95% CI: 0.53–0.97, p = 0.031).

**Table 4:** Multivariable-adjusted ORs and 95%CI of the SUA/Cr associated with TVEs in DM patients.

	Number of TVEs	Unadjusted OR (95% CI)	Unadjusted *P*-value	Adjusted OR (95% CI)	Adjusted *P*-value
Model1	35	0.7 (0.53~0.94)	0.016	0.72 (0.53~0.97)	0.031
Model2	35	0.7 (0.53~0.94)	0.016	0.72 (0.53~0.97)	0.03
Model3	35	0.7 (0.53~0.94)	0.016	0.73 (0.54~0.99)	0.04
Model4	35	0.7 (0.53~0.94)	0.016	0.72 (0.53~0.97)	0.031

OR, odds ratio; CI, confidence interval.Model1: adjusted for Gender, Previous stroke, Previous PCI.

Model2: model1+further adjusted for SBP, TG.

Model3: model2+further adjusted for 2-diseased vessels, ≥3-diseased vessels, LM, LCX.

Model4: model3+further adjusted for bifurcation lesion, restenosis, length of stent, diameter of stent, Age.

**Figure 2:**
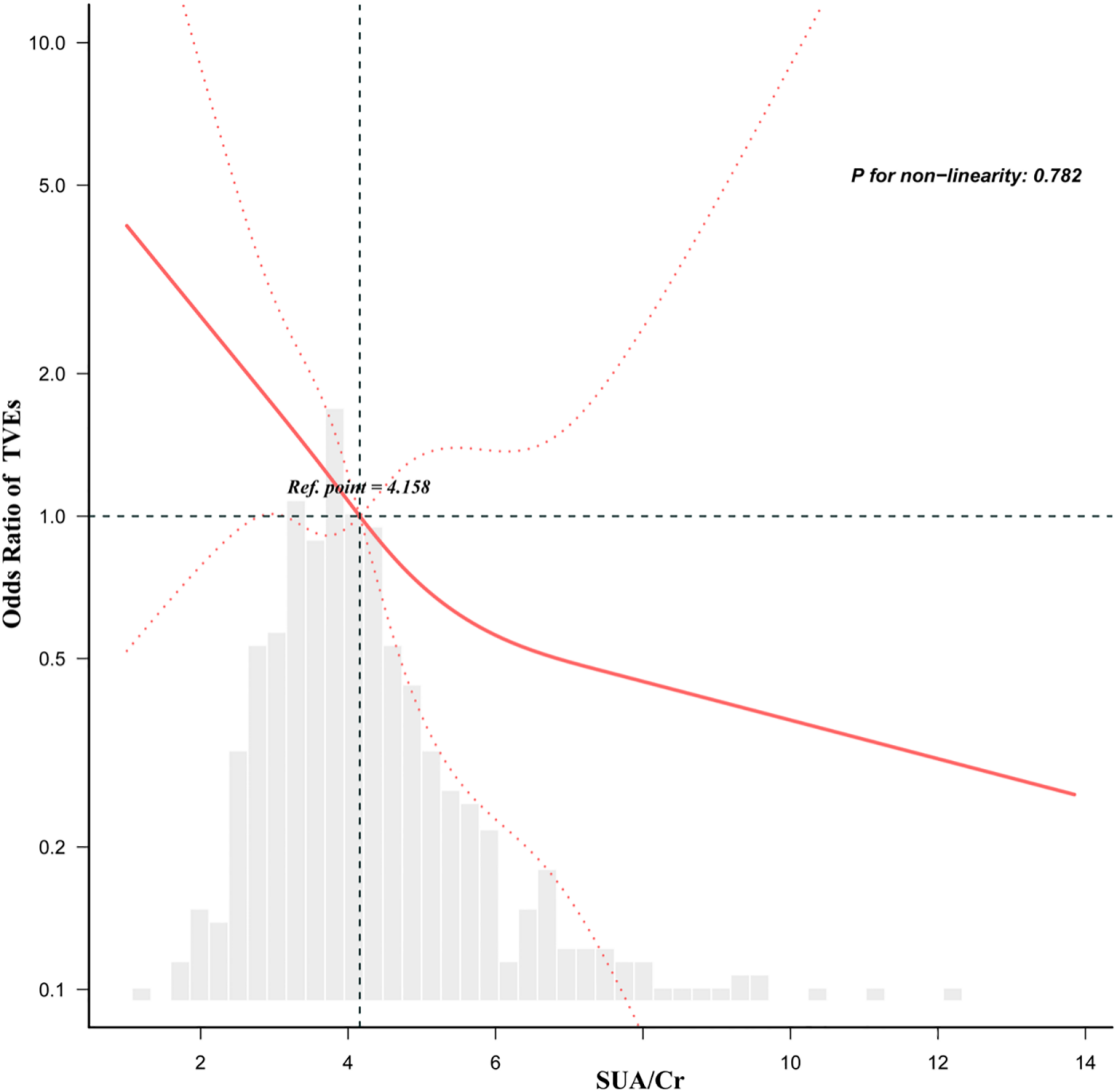
Dose-response relationship between the SUA/Cr and TVEs in DM patients adjusted for model 4. Gender, Previous stroke, Previous PCI, SBP, TG, 2-diseased vessels, ≥3-diseased vessels, LM, LCX, bifurcation lesion, restenosis, length of stent, diameter of stent, Age. All of the datas are displayed. Odds ratios are indicated by solid lines and 95% CIs by dashed areas.

The dose-response analysis, adjusted for variables in Model 4, revealed a linear inverse relationship between SUA/Cr and the risk of TVEs in DM patients (**Figure 2**). Restricted cubic spline analysis confirmed this linear and negative association, with a threshold (knot) at 4.158 (p for non-linearity = 0.782).

In DM patients, subgroup analysis revealed that SUA/Cr was significantly associated with a lower risk of TVEs among non-smokers (adjusted OR = 0.68, 95% CI: 0.47–0.98, p = 0.041) and those presenting with NSTE-ACS (adjusted OR = 0.54, 95% CI: 0.34–0.85, p = 0.008) (**Table 5**). However, no significant associations were observed in other subgroups, including smokers, STEMI patients, or those with SA (all p for interaction > 0.05).

**Table 5:** Subgroup analysis on the association of SUA/Cr with TVEs in the DM patients.

Subgroups	n total	n event(%)	Unadjusted OR(95% CI)	Unadjusted *P*-value	Adjusted OR(95% CI)	Adjusted *P*-value	P for interaction
Smoking							0.564
No	344	26 (7.6)	0.65 (0.46~0.93)	0.017	0.68 (0.47~0.98)	0.041	
Yes	104	9 (8.7)	0.84 (0.51~1.39)	0.501	0.84 (0.44~1.63)	0.611	
Clinical presentation							0.021
STEMI	111	3 (2.7)	1.29 (0.87~1.9)	0.202	3.44 (0.65~18.17)	0.145	
NSTE-ACS	275	25 (9.1)	0.55 (0.37~0.81)	0.002	0.54 (0.34~0.85)	0.008	
SA	62	7 (11.3)	0.65 (0.31~1.38)	0.267	0.32 (0.09~1.16)	0.082	
eGFR							0.612
<90	106	12 (11.3)	0.65 (0.34~1.25)	0.199	0.68 (0.32~1.43)	0.308	
≥90	342	23 (6.7)	0.75 (0.53~1.06)	0.1	0.76 (0.53~1.11)	0.161	
Gender							0.314
Female	172	11 (6.4)	0.59 (0.35~0.98)	0.041	0.61 (0.34~1.09)	0.093	
Male	276	24 (8.7)	0.79 (0.55~1.13)	0.193	0.84 (0.57~1.24)	0.389	
Age							0.791
≥60	277	21 (7.6)	0.69 (0.47~1.02)	0.064	0.68 (0.44~1.05)	0.085	
<60	171	14 (8.2)	0.7 (0.45~1.08)	0.109	0.81 (0.52~1.27)	0.362	

OR, odds ratio; CI, confidence interval; eGFR, estimated glomerular filtration rate.

## 4 Discussion

In this study, we found that a higher SUA/Cr ratio was independently associated with a significantly lower risk of TVEs in DM patients. Notably, SUA/Cr exhibited a linear inverse relationship with TVEs risk, with a threshold identified at 4.158, suggesting that the risk continuously declines as SUA/Cr levels increase. Subgroup analysis further demonstrated that this protective effect was particularly evident in non-smokers and DM patients with NSTE-ACS. These findings underscore the potential of SUA/Cr as a prognostic biomarker for DM patients undergoing PCI, providing valuable insights for risk stratification and long-term management strategies. To the best of our knowledge, this is the first study to systematically examine the association between SUA/Cr and long-term adverse target vessel outcomes in DM patients post-PCI. Unlike previous research, which has largely overlooked procedural cardiovascular outcomes such as TVEs or stent-related complications, our study addresses this gap. Moreover, earlier studies often lacked comprehensive subgroup stratification or dose–response analyses, limiting their clinical relevance. By employing advanced modeling approaches in a well-defined post-PCI diabetic cohort, our study overcomes these limitations and enhances the applicability of the findings.

Hyperuricemia has long been recognized as a condition closely linked to cardiovascular disease (CVD) and is considered a potential cardiovascular risk factor (26). Elevated SUA levels have been associated with an increased risk of CAD, hypertension, heart failure, and chronic kidney disease (27), with potential underlying mechanisms involving oxidative stress, inflammation, and endothelial dysfunction (8). However, recent studies suggest that SUA may have a dual role in cardiovascular health, where moderately elevated levels could confer protective effects, while both excessively high and low SUA levels may contribute to a greater CVD risk (28). Notably, SUA exhibits antioxidant properties under certain conditions, particularly in maintaining vascular homeostasis. As a free radical scavenger, it can mitigate oxidative stress-induced vascular injury and provide endothelial protection (9–11).

Lazzeri et al. reported that elevated SUA levels were associated with higher in-hospital mortality in patients with STEMI, independent of Killip classification (5). Similarly, Li et al. identified hyperuricemia as an independent risk factor for short-term adverse cardiovascular events in STEMI patients (6). However, Liu et al. found no significant association between SUA levels and mortality risk in STEMI patients with Killip class II-IV (7). Additionally, some studies have suggested that SUA is not an independent predictor of cardiovascular events or all-cause mortality in patients with type 2 diabetes (29).These discrepancies may stem from the influence of renal clearance on SUA levels, as evaluating SUA in isolation may be confounded by renal function status, thereby limiting its accuracy as a prognostic indicator (12). To address this issue, the SUA/Cr has been introduced as a composite biomarker that accounts for both SUA and renal function. Emerging evidence suggests that SUA/Cr provides a more accurate reflection of metabolic abnormalities and serves as a more reliable predictor of metabolic syndrome compared to SUA or creatinine alone (30).

In recent years, accumulating evidence has underscored a strong association between the SUA/Cr and adverse cardiovascular events. However, its precise role remains controversial, as some studies identify SUA/Cr as an independent risk factor for cardiovascular complications, while others report conflicting findings. A nationwide multicenter study from Italy demonstrated that SUA/Cr independently predicted cardiovascular events in DM patients (21). Further investigations revealed a nonlinear association between SUA/Cr levels and cardiovascular mortality risk in this population. Specifically, among DM patients with preserved renal function, the risk of cardiovascular mortality increased significantly when SUA/Cr exceeded 5.35, whereas in those with impaired renal function, the threshold for heightened risk was SUA/Cr >7.5 (12).Additionally, Zeng et al. reported a U-shaped nonlinear relationship between SUA/Cr and all-cause mortality in hypertensive patients, with a critical turning point at 4.3 (31). Similarly, Zhang YD et al. found that elevated preoperative SUA/Cr levels were significantly associated with a higher risk of atrial fibrillation (AF) recurrence following catheter ablation, suggesting that SUA/Cr may serve as a novel prognostic biomarker for AF recurrence (32).

Notably, the relationship between SUA/Cr and cardiovascular outcomes may vary across different populations. Tang Z et al. reported a linear inverse association between SUA/Cr and cardiovascular mortality in hypertensive patients, with a continuous decline in mortality risk as SUA/Cr increased (P for nonlinearity = 0.32) and a threshold identified at 6.54 (13). Similarly, Jiang L et al. found that lower SUA/Cr levels were associated with an increased risk of in-hospital adverse cardiovascular events in elderly patients with AMI, with this association being more pronounced in males (14).However, studies investigating the prognostic value of SUA/Cr in post-PCI outcomes remain limited. In the present study, we found that higher SUA/Cr levels were significantly associated with a lower risk of TVEs in DM patients following PCI, and this association remained robust after adjusting for multiple confounders (Gender, Previous stroke, Previous PCI, SBP, TG, 2-diseased vessels, ≥3-diseased vessels, LM, LCX, bifurcation lesion, restenosis, length of stent, diameter of stent, Age).The observed protective effect may be attributed to the antioxidant and anti-inflammatory properties of SUA. DM patients are typically in a state of chronic low-grade inflammation and oxidative stress, both of which contribute to coronary artery remodeling and an increased risk of in-stent restenosis following PCI (33). Emerging evidence suggests that in DM patients, dysregulated uric acid metabolism may serve as a compensatory mechanism to counteract oxidative stress-induced vascular damage (34). Moderately elevated SUA levels may function as an endogenous antioxidant, scavenging free radicals, mitigating endothelial dysfunction, and inhibiting low-density lipoprotein oxidation, thereby slowing the progression of atherosclerosis and potentially reducing the risk of TVEs (35–37).

These findings, supported by biological plausibility, suggest that SUA/Cr may serve as a compensatory antioxidant factor in specific clinical contexts, particularly in diabetic patients post-PCI. However, the relationship between SUA/Cr and cardiovascular outcomes remains inconsistent across studies. For instance, while the URRAH project (12), reported a U-shaped association between SUA/Cr and cardiovascular mortality, our study demonstrated a linear inverse relationship with target vessel events in PCI-treated diabetic patients. These discrepancies likely arise from key methodological and clinical differences. First, outcome definitions varied significantly: previous studies primarily examined all-cause or cardiovascular mortality, whereas we focused on procedural outcomes (target vessel revascularization and in-stent restenosis). Second, our study population was strictly limited to PCI-treated CAD patients with diabetes, unlike broader community-based cohorts in prior research. Third, earlier studies frequently omitted adjustments for critical angiographic and procedural variables (e.g., stent diameter, lesion complexity, or prior PCI history) that may influence the SUA/Cr-TVE relationship. Finally, while some studies categorized SUA/Cr into quantiles assuming nonlinearity, we employed restricted cubic spline models to objectively confirm a consistent linear inverse association. These collective differences underscore how clinical context and study design fundamentally shape the interpretation of SUA/Cr’s prognostic value.

Furthermore, our study demonstrated that the association between SUA/Cr and TVEs risk was particularly pronounced in non-smokers and DM patients with NSTE-ACS. Specifically, higher SUA/Cr levels were significantly correlated with a lower risk of TVEs in non-smokers, while this protective effect was even more pronounced in NSTE-ACS patients. This differential effect may be attributed to variations in oxidative stress burden and systemic inflammation. In non-smokers, relatively lower oxidative stress and inflammatory activity may allow the antioxidant and endothelial-protective properties of uric acid to exert greater benefits (38). Additionally, unlike STEMI, where ischemic events are predominantly driven by acute thrombotic occlusion, NSTE-ACS is characterized by chronic inflammation and atherosclerotic plaque instability, which play a central role in disease progression (39). In this context, uric acid, functioning as an endogenous antioxidant, may contribute to plaque stabilization and attenuation of inflammation-mediated endothelial dysfunction, thereby mitigating the risk of TVEs.

While some studies, such as those from the URRAH project (12), reported a U-shaped or nonlinear association between SUA/Cr and cardiovascular mortality, our study identified a linear inverse relationship between SUA/Cr and target vessel events in diabetic patients undergoing PCI. These differences may stem from multiple factors. First, the outcome definitions differ: prior studies focused on all-cause or cardiovascular mortality, whereas we specifically assessed target vessel revascularization and in-stent restenosis—which are procedural and lesion-level outcomes. Second, our study population consisted entirely of PCI-treated CAD patients, with a defined post-intervention context, while others included broader community or hospital cohorts. Third, previous studies often did not adjust for detailed angiographic and procedural characteristics such as stent diameter, lesion complexity, or prior PCI, which may confound the SUA/Cr–TVE relationship. Finally, some earlier studies categorized SUA/Cr into quantiles and assumed nonlinear relationships, whereas our study confirmed a linear trend using restricted cubic spline analysis. These methodological and population-level distinctions may explain the observed differences and suggest that the prognostic significance of SUA/Cr may be context-dependent—particularly relevant for stent-related vascular outcomes in diabetic populations.

There are several limitations to this study. First, as a retrospective single-center study, it is subject to selection bias and information bias, which may limit the external validity of our findings. Although the sample size was relatively large (n = 2087), further validation through large-scale, multicenter studies is required to enhance the robustness of our results. Second, this study only established an association between SUA/Cr and post-PCI TVEs, precluding any inference of causality. Future prospective, multicenter, and long-term follow-up studies are warranted to further elucidate the prognostic value of SUA/Cr and its potential role in post-PCI risk stratification and clinical management.

## 5 Conclusion

This study found that higher SUA/Cr levels were associated with a lower risk of TVEs in DM patients following PCI, suggesting that SUA/Cr may serve as a biomarker for cardiovascular risk assessment. Given its ease of measurement and cost-effectiveness, SUA/Cr has the potential to facilitate early risk identification and personalized management in high-risk post-PCI patients, thereby improving clinical outcomes. However, its clinical utility requires further validation. Future large-scale prospective studies are needed to establish the definitive role of SUA/Cr in this patient population and to explore its potential applications in routine cardiovascular risk stratification.

## Data Availability

The datasets presented in this study can be found in online repositories. The names of the repository/repositories and accession number(s) can be found in the article/**Supplementary Material**.
